# Voluntary wheel running promotes myelination in the motor cortex through Wnt signaling in mice

**DOI:** 10.1186/s13041-019-0506-8

**Published:** 2019-10-24

**Authors:** Jian Zheng, Xuan Sun, Chaolin Ma, Bao-ming Li, Fei Luo

**Affiliations:** 1 Institute of Life Science, Nanchang University, Nanchang, 330031 China; 2 School of Basic Medical Sciences, Nanchang University, Nanchang, 330006 China

**Keywords:** Voluntary running exercise, Oligodendrocyte, Myelination, Motor functions, Wnt signaling, Mice

## Abstract

Myelin of the central nervous system exhibits strong plasticity, and skill learning exercise promotes oligodendrogenesis and adaptive myelination. Increasing evidence shows that brain structures and functions are affected by physical activity. However, the impact of voluntary physical activity on central myelination and its underlying mechanism remains unclear. The present study aimed to investigate the effect of voluntary wheel running (VWR) on central oligodendrogenesis and adaptive myelination in mice. Adult C57BL/6 J mice were placed in running wheels and allowed for voluntary running 2 weeks. Myelin levels in the central nervous system were detected using western blotting, qRT-PCR, immunohistochemical staining, and electron microscopy. Oligodendrocyte precursor cells (OPCs) and oligodendrocytes (OLs) were detected using immunohistochemical staining and 5-bromo-2-deoxyuridine (BrdU) assays. Motor abilities of the animals were examined using open-field, rotarod running, and beam-walking behavioral paradigms. Vital molecules of Wnt signaling were detected, and the involvement of such molecules was verified using in vitro culture of OPCs. Our results showed that VWR significantly enhanced the myelination in the motor cortex. VWR promoted the proliferation and differentiation of OPCs, and the maturation of OLs. The VWR-regulated myelination was associated with the improved motor skill and decreased mRNA level of Wnt3a/9a, whereas stimulation of Wnt signaling pathway with Wnt3a or Wnt9a suppressed OPCs proliferation and differentiation in vitro. The present study demonstrated that physical activity is highly efficient at promoting myelination in the motor cortex, by enhancing the proliferation of OPCs and accelerating the generation of myelin, providing a step forward in understanding the beneficial effects of physical activity on central myelination and its underlying mechanism.

## Introduction

Myelin, the multi-laminar sheath that surrounds and insulates axons in the central nervous systems (CNS) and is formed by multipolar glial cells called oligodendrocytes (OLs), greatly facilitates the rapid transmission of neural impulses [1–3]. In humans and non-human primates, myelination persists throughout adulthood in the CNS and involves the generation of new myelinating OLs [3, 4]. Such prolonged period of myelin development opens a window for individual experiences to influence the myelination [5, 6]. Compelling evidence indicates that a widespread proliferating population of glial antigen-2 (NG2) or platelet derived growth factor receptor alpha (PDGFRα) positive cells, termed NG2-glia or oligodendrocyte precursor cells (OPCs), are the major source of newly generated mature OLs required for myelination [7, 8].

Physical activity has beneficial effects on the health of the CNS, and is of great importance for the nervous system to resist diseases and injuries [9]. Previous studies have focused on the changes of various nerve growth factors after exercise and the effects of exercise on neurogenesis [10, 11]. It is reported that running could promote neurogenesis in the hippocampus [12, 13], and partially reverse aging-associated decline in synapses and neurogenesis [14–16]. Running also enhances synaptic transmission and plasticity, cellular activity in the hippocampus [10, 11, 17].

However, it is unclear whether physical activity could produce beneficial effects on CNS myelination and promotes motor functions, and if so, what is the underlying mechanism? To address this question, the present study investigated the impact of voluntary wheel running (VWR) on the myelination of the CNS, especially in the motor cortex. We demonstrated that VWR significantly enhances the myelination and motor coordination ability via inhibiting Wnt signaling in oligodendroglial lineage cells.

## Methods

### Experimental animals

C57BL/6 J male mice (8-week old) were kept on a 12 h light/dark cycle in a temperature-controlled room (25 °C). All mice were individually housed in a light and humidity controlled climatic chamber (SPF condition), fed with HEPA-filtered air, and provided with irradiated food and water. Mice were provided with a regular chow diet (Lab Diets, #5001). For voluntary-wheel running, mice were housed in cages containing a 5-in. running wheel. Wheel running was entirely voluntary, and no means were used to promote or ensure activity [18]. Control mice were housed in similar cages except that the wheel was locked. All animal procedures were carried out in accordance with the principles of laboratory animal care and use approved by the Nanchang Animal Care and Use Committee guidelines.

### Protein extraction and western blotting

Lysates were generated using RIPA buffer (Thermo Scientific) supplemented with 1% protease inhibitor cocktail set III EDTA-free (vol/vol, Calbiochem). Samples were heated at 100 °C for 10 min, and 10 μg of total protein was loaded onto 12% acrylamide gel. Proteins were then transferred onto polyvinylidene difluoride (PVDF) membranes (Millipore). Membranes were blocked with 5% powdered milk in Tris-buffered saline (TBST) for 2 h at room temperature on an orbital shaker, and incubated overnight at 4 °C with primary antibody (see Additional file 1: Table S1 for detail), then washed thrice in TBST for 5 min, and incubated with horseradish peroxidase (HRP)-conjugated IgG secondary antibody (see Additional file 2: Table S2 for details) for 2 h at room temperature. Chemiluminescent substrate detection using reagent RapidStep ECL Reagent (Thermo) and autoradiography film processing was performed, followed by analysis with Image J (NIH, http://imagej.nih.gov/ij).

### Quantitative real-time PCR (qRT-PCR) analysis

Total RNA was isolated from snap-frozen brain tissue using Trizol (Invitrogen) according to the manufacturer’s protocol. First strand cDNA was synthesized from total RNA using primers and SuperScriptIII reverse transcriptase (Invitrogen). Quantitative real-time PCR (qRT-PCR) was carried out using the ABI Prism 7700 Sequence Detection System and SYBR Green Master Mix according to the manufacturer’s directions (Applied Biosystems). Relative mRNA expression levels were calculated via a comparative threshold cycle (C_t_) method using GAPDH as an internal control: △Ct = C_t_ (gene of interest) - C_t_ (GAPDH). Gene expression fold change was normalized to control sample and then calculated as 2^-△Ct^. Primers used for qRT-PCR were listed in Additional file 3: Table S3.

### Immunohistochemistry and laser confocal scanning

Mice were administered ketamine and xylazine (150 mg/kg and 10 mg/kg, respectively) and perfused with 0.1 M PB followed by ice-cold 4% paraformaldehyde (PFA). Brains were postfixed in 4% PFA overnight at 4 °C. Brain tissues were cryoprotected in 10, 20, 30% (w/v) sucrose (Sigma) before frozen in optimal cutting temperature compound. Next, Brain slices (30 μm in thickness) were collected and processed as floating slices. Primary antibodies (see Additional file 1: Table S1 for details), the immunoreactivity of which was determined before use, were diluted in blocking solution (0.1% [v/v] Triton X-100 and 10% fetal calf serum in 0.01 M PBS) and applied to slices overnight at 4 °C. Negative controls were performed by replacing the primary antibodies with normal rabbit serum. On the next day, brain slides were washed 3 times with PBS and incubated for 2 h with secondary antibodies (see Additional file 2: Table S2 for details). Finally, brain slices were mounted on slides. Mounted slides were imaged using an inverted laser confocal microscope (FV1000; Olympus). Cells were counted using FV10-ASW (Olympus) and Image J (NIH, http://imagej.nih.gov/ij).

### 5-bromo-2-deoxyuridine (BrdU) assays

For cumulative labeling experiments, BrdU (Sigma) was intraperitoneally injected once a day for 2 weeks. Brain slices (30 μm in thickness) were prepared as described above. Brain slices were then washed in PBS and incubated in 0.2% Triton X-100 in PBS for 1 h at 20–25 °C. DNA was denatured by two successive incubation steps (10 min each step in 2 N HCl) and neutralized by two successive incubations (10 min each step in 0.1 M sodium tetraborate with pH 8.5). Then, immunohistochemistry and laser confocal scanning were conducted as described above.

### Expression of Wnt3a and Wnt9a in HEK293 cells

Expression of Wnt3a and Wnt9a in HEK293 cells was performed as described previously [19]. In brief, well-prepared pcDNA3-FLAG-Wnt3a/Wnt9a or p3xFLAG vector plasmid were transfected into HEK293T cells by polyethylenimine (PEI). 6–8 h later, the cultured medium was changed with free-serum conditional medium and left overnight. Then, the cell lysis and part of conditional medium were used to detect the proteins of Wnts by western blotting.

### Culture of OPCs and Wnt3a/9a treatment

Oligodendrocyte precursor cells (OPCs) were cultured according to the previous method [20], but with a little modification. Briefly, brain cortical tissues was isolated from postnatal 2-day’s rat pups and dissociated by trypsin (0.25%). Cortical cells were plated in PDL (0.1 mg/ml) pre-coated T25 flask incubator with 5% CO_2_ at 37 °C. OPCs were isolated and cultured in serum-free oligodendrocyte growth medium supplemented with bFGF and PDGF-AA. The conditional medium containing previously-collected Wnts were added into the growth medium. Cortical cells were treated for 4 days in 37 °C incubator containing 5% CO_2_, fixed and finally immunohistochemically stained as described above. As the number of cortical OPCs was more, and the status of the OPCs was better in rat than in mouse, we therefore used rat instead of mouse for this experiment.

### Electron microscopy investigation

Mice were perfused with 0.1 M PB followed by 2% gluteraldehyde/4% PFA in sodium cacodylate buffer. A block of tissue (approximately 1 × 1 × 2 mm^3^) from the motor cortex was dissected and postfixed overnight at 4 °C. Brain tissue was dehydrated in an ethanol gradient from 50% through 100%, and embedded in Epon. Brain tissue was cut into ultra-thin slices (70 nm in thickness). Brain slices were stained with 2% uranyl acetate (v/v) and Reynolds lead citrate, and examined with a transmission electron microscope (Hitachi) [21]. The G-ratio, which reflects the thickness of myelin, was determined using the formula as follows: G-ratio = [axonal diameter (without myelin sheath) ÷ fiber diameter (axon+myelin sheath)].

### Open-field test

Mice were habituated in the experimental room for 1 h prior to test. Open-field test was used to assess gross locomotion ability [8]. Briefly, each mouse was placed in a 45× 45 cm^2^ open-field arena, and its locomotion activities was recorded for 10 min using video capture software. Total locomotion distance and velocity were measured. The open field arena was cleaned with 70% ethanol before used for the next animal.

### Rotarod-running test

We used the rotarod to assess the sensorimotor balance and coordination abilities of mice as described previously [22, 23]. Briefly, mice were pre-trained on the rod at 10 rpm for 5 min on Day 1. On the next day, mice were placed on the rod and the rotating velocity of the rod was set at 4 rpm at the very beginning, and accelerated to 40 rpm in 300 s. The test included 4 trials in total, with an inter-trial interval of 5 min. The time to fall down from the rotating rod was recorded for each mouse.

### Beam-walking test

Beam-walking test was a second paradigm assessing the sensorimotor balance and coordination ability [22]. Mice were required to walk on a square wood beam with 6 mm in width, 80 cm in length and 30 cm apart from the ground. Mice were pre-trained to walk on the beam. On the next day, the formal training was performed and include 3 trials. Each trial started by placing mice on one end of the beam. Mice were required to walk to the other end of the beam, and to enter into a black box (safe place). The total number of foot slips during the beam-walking process was collected.

### Statistical analysis

All statistical analyses including testing the normality of data distribution were performed using GraphPad Prism 6 and *p* value < 0.05 was considered as significant difference. For comparing difference between 2 groups with normally distributed datasets, unpaired Student’s t-test was used. Correlation analysis were assessed using Pearson’s rank correlation test.

## Results

### VWR promotes myelination in the motor cortex

**Fig. 1:**
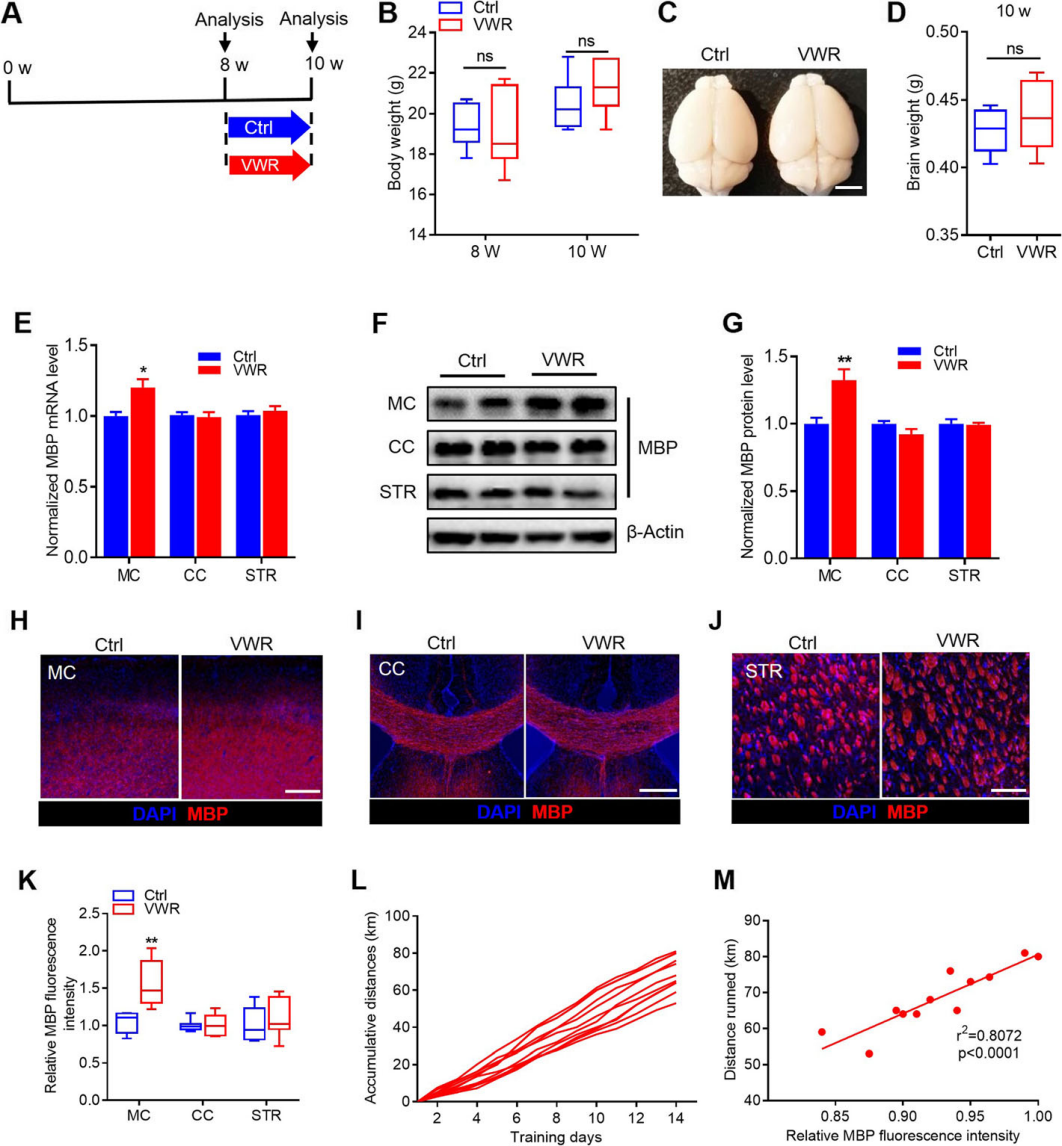
Voluntary wheel running (VWR) accelerates myelination in the motor cortex. **a** Time course schema for animal treatment and testing. **b** Body weight of Control (Ctrl) and VWR mice. *n* = 7 per group. ns, *p* > 0.05, unpaired Student’s t-test. **c** Brains removed from Ctrl and VWR mice. Scale bar = 5 mm. **d** Brain weight of Ctrl and VWR mice. *n* = 7 per group. ns, *p* > 0.05, unpaired Student’s t-test. **e** mRNA levels of *MBP* in different brain regions of Ctrl and VWR mice. *n* = 6 per group. ^*^p < 0.05 vs Ctrl, unpaired Student’s t-test. **f, g** Protein levels of MBP in different brain regions of Ctrl and VWR mice. *n* = 6 per group. ^**^p < 0.01 vs Ctrl, unpaired Student’s t-test. **h-j** Confocal images of MBP staining in different brain regions of Ctrl and VWR mice. Scale bar = 500 μm. **k** Quantitative analysis of the relative MBP fluorescent intensity in different brain regions of Ctrl and VWR mice. *n* = 7 per group. ^**^p < 0.01 vs Ctrl, unpaired Student’s t-test. **l** Accumulative running distances on the wheel of individual mice with training. **m** Correlation analysis between relative MBP fluorescence intensity in the motor cortex and running distances. Data are presented as mean ± SEM or minimum to maximum. MC, motor cortex; CC, corpus callosum; STR, striatum

To administer voluntary wheel running (VWR), we used a voluntary running task in which mice were given unrestricted access to a monitored running wheel for 2 weeks (Fig. 1a). VWR mice were individually housed in modified cages, with each cage containing a 5-in. running wheel. Control mice were housed in an identical setting, with a locked wheel. After 2-week voluntary running, the VWR mice exhibited a similar body weight and brain weight with the control mice (Fig. 1b-d).

To evaluate the effect of VWR on myelination, we first examined the mRNA and protein levels of myelin basic protein (MBP) in three different brain regions related to motor function. As shown in Fig. 1e-g, the VWR mice demonstrated a significant increase in the mRNA and protein levels of MBP in the motor cortex, but not in the corpus callosum and striatum relative to controls. Consistently, immunohistochemistry for MBP showed that VWR mice displayed a markedly enhanced myelination in the motor cortex, but not in the corpus callosum and striatum relative to controls (Fig. 1h-k). Regression analysis revealed a significant correlation between running distance with relative MBP fluorescence intensity in the motor cortex in individual VWR mice (Fig. 1l and m). This result indicates that VWR could promote the myelination of the motor cortex.

**Fig. 2:**
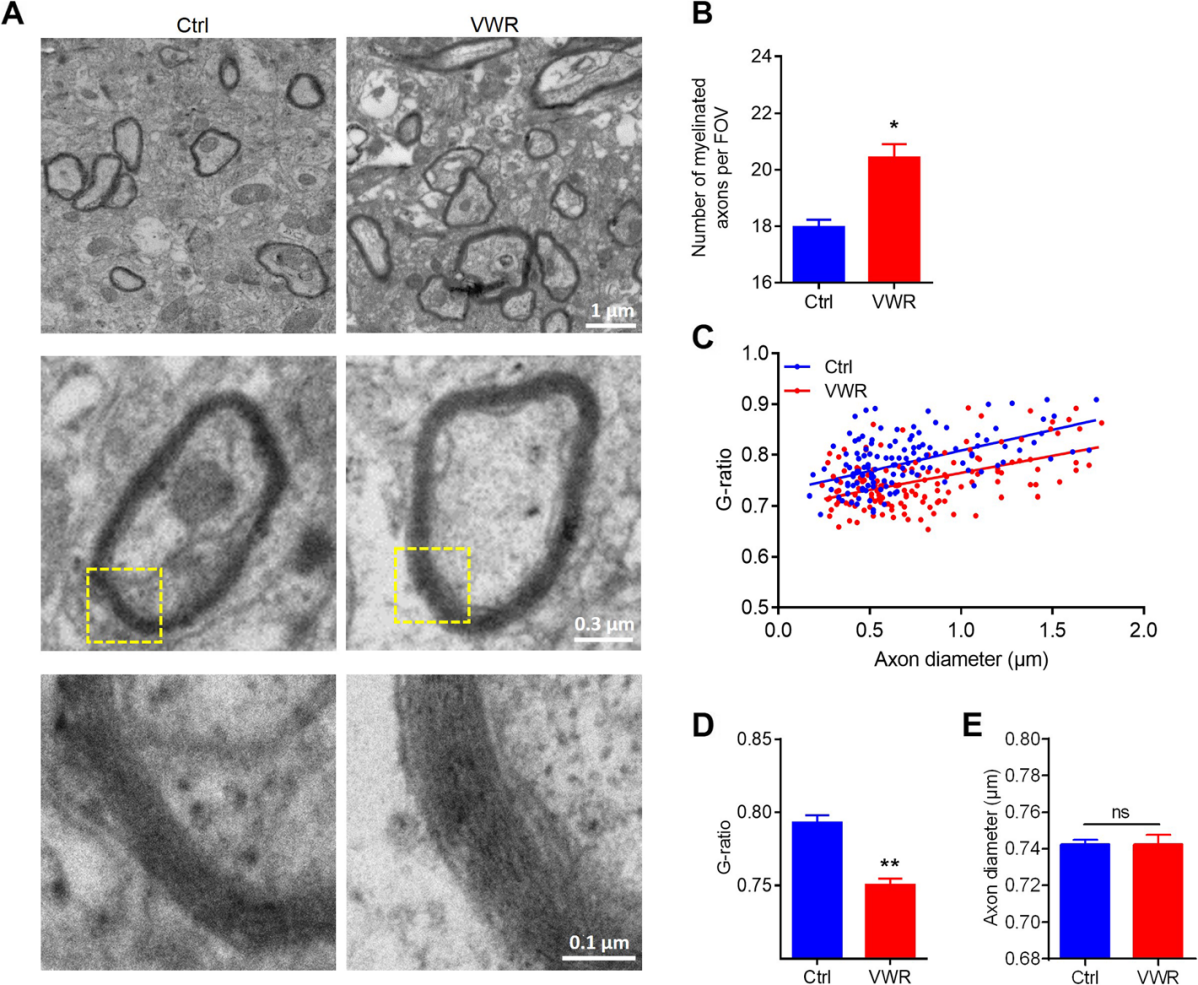
Voluntary wheel running (VWR) increases the number and the thickness of myelinated axons in the motor cortex. **a** Representative electron micrographs of axon and myelin in control (Ctrl) and VWR mice. **b** Quantification of myelinated axons per field of view (FOV = 506 μm^2^) in Ctrl and VWR mice. **c, d** G-ratio analysis for the thickness of myelin in Ctrl and VWR mice. **e** Diameter of axons in Ctrl and VWR mice. For electron microscopy, enough independent fields were quantified to achieve at least 150 axons per group. Data are presented as mean ± SEM. *n* = 150 from3 mice per group. ^*^p < 0.05 vs Ctrl; ^**^p < 0.01 vs Ctrl; ns, *p* > 0.05, unpaired Student’s t-test and Pearson’s rank correlation test

To further confirm that VWR could promote myelination, we performed electron microscopy analysis in the motor cortex (Fig. 2a). A significant increase in the number of myelinated axons (Fig. 2b) and the thickness of myelin was observed in the motor cortex of the VWR mice, as evidenced by the decreased average G-ratio (Fig. 2c, d), while the diameter of axons kept unchanged (Fig. 2e).

Taken together, these results provide solid evidence for a substantial beneficial effect of VWR on promoting myelination in the motor cortex.

### VWR enhances OPCs proliferation and differentiationin the motor cortex

**Fig. 3:**
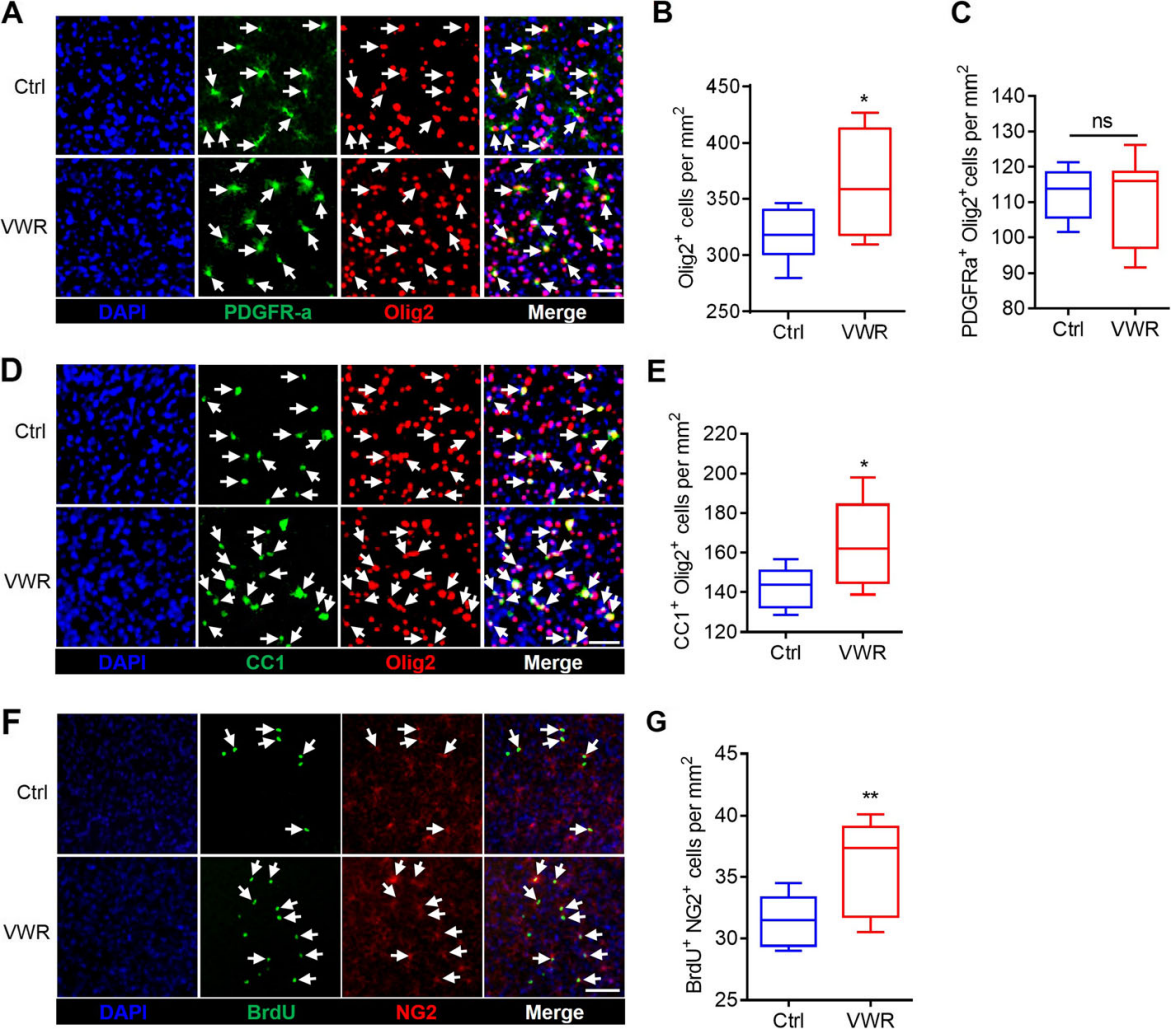
Voluntary wheel running (VWR) accelerates OPC proliferation and OL maturation in the motor cortex. **a** Confocal images of PDGFRα- and Olig2-possive cells in the motor cortex of control (Ctrl) and VWR mice. Scale bar = 50 μm. **b** VWR mice have significantly more oligodendroglial lineage cells (Olig2^+^) than Ctrl mice. **c** VWR mice have statistically equal number of OPCs (PDGFRα^+^Olig2^+^) as control mice. **d** Confocal images of CC1- and Olig2-positive cells in the motor cortex of Ctrl and VWR mice. Scale bar = 40 μm. **e** VWR mice have significantly more mature OLs (CC1^+^Olig2^+^) than Ctrl mice. **f** Confocal images of BrdU- and NG2-positive cells in the motor cortex of Ctrl and VWR mice. Scale bar = 60 μm. **g** VWR mice have significantly more newly generated OPCs (NG2^+^ BrdU^+^) than Ctrl mice. Data are presented as minimum to maximum. *n* = 8 per group. ^*^p < 0.05 vs Ctrl; ns, *p* > 0.05;^**^p < 0.01 vs Ctrl; unpaired Student’s t-test

OPCs (PDGFRα^+^ Olig2^+^) arise from neuroepithelial progenitors in the ventral neural tube, and they migrate, proliferate, and ultimately differentiate to form mature OLs (CC1^+^ Olig2^+^) [24–27]. Thus, we analyzed the density of OPCs and OLs in the motor cortex by immunohistochemistry. As shown in Fig. 3, the density of OPCs was not affected by VWR (Fig. 3a, c), while oligodendroglial lineage cells (Olig2^+^) and mature OLs significantly increased in VWR mice (Fig. 3b, d and e). To determine if OPCs proliferation was up-regulated, we examined the profile of OPCs proliferation. Mice were administered BrdU from 8- to 10-week old (once a day), and cumulative BrdU incorporation was measured at 10-week old. A significant increase of newly generated OPCs (BrdU^+^NG2^+^) was observed in VWR mice (Fig. 3f and g). Thus, VWR could promote OPCs proliferation and dramatically increase the number of mature OLs in the motor cortex.

### VWR promotes motor coordination performance

**Fig. 4:**
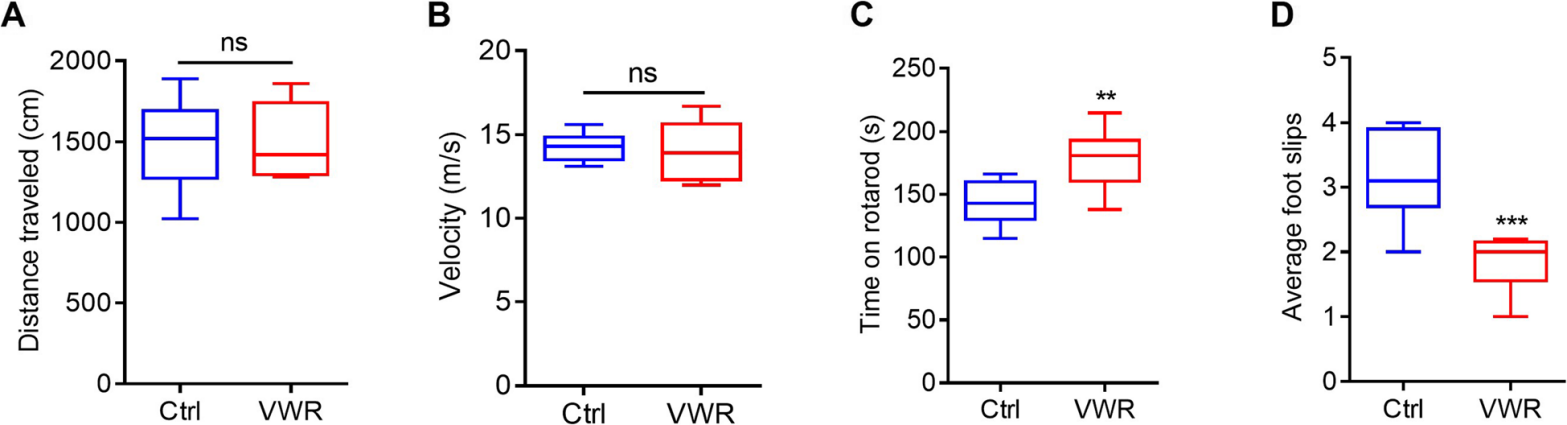
Voluntary wheel running (VWR) promotes motor performance in mice. **a** Total distance traveled in the open field test for control (Ctrl) and VWR mice. **b** The average locomotion speed in the open field test. **c** Time on the rotating bar in the rotarod test. **d** Average foot slips in the beam-walking test. Data are presented as minimum to maximum. *n* = 9 per group. ns, *p* > 0.05; ^**^p < 0.01 vs Ctrl; ^***^p < 0.001 vs Ctrl; unpaired Student’s t-test

To evaluate motor ability change associated with VWR-enhanced oligodendrogenesis and myelination in the motor cortex, we measured gross motor function and fine motor coordination ability using open-field, rotarod-running and beam-walking tests. As shown in Fig. 4, the total distance travelled and the average locomotion speed in the open field test were not changed in the VWR mice (Fig. 4a and b). However, the VWR mice ran significantly longer in the rotating rotarod, and displayed less foot slips in the beam-walking test relative to the controls (Fig. 4c, d). This result suggest that VWR could promote fine motor coordination ability.

### VWR promotes myelination via Wnt signaling pathway

**Fig. 5:**
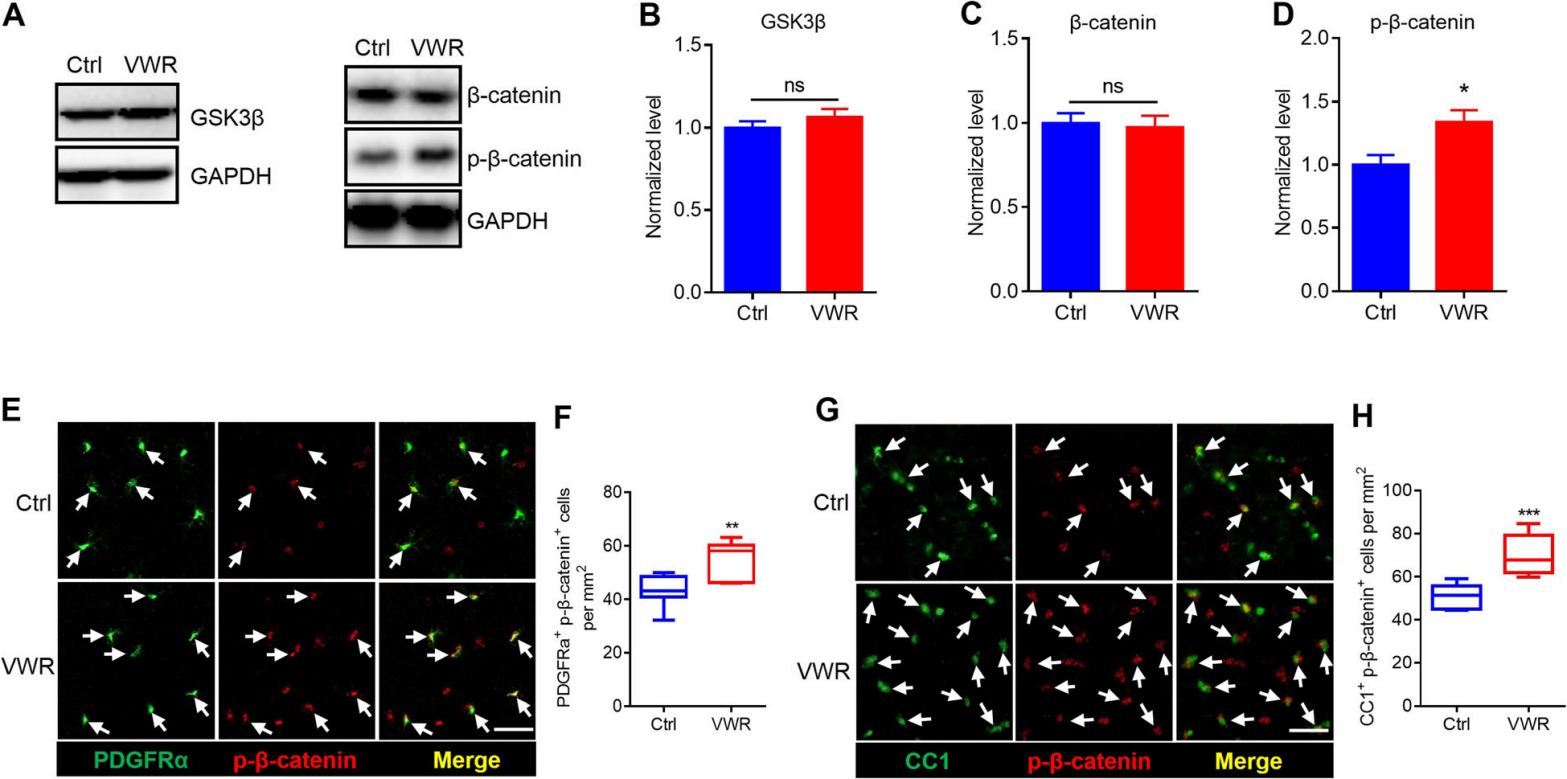
Voluntary wheel running (VWR) inhibits Wnt signaling. **a**-**d** Western blotting analysis of GSK-3β, β-catenin and p-β-catenin in the motor cortex (MC) of control (Ctrl) and VWR mice. As shown, p-β-catenin, but not GSK-3β and β-catenin, was up-regulated in the VWR mice. Data are presented as mean ± SEM. *n* = 6 per group. ns, *P* > 0.05; ^*^p < 0.05 vs Ctrl; unpaired Student’s t-test. **e**, **f** Confocal images and statistic histograms illustrating the co-localization of p-β-catenin with PDGFRα. As shown, VWR mice have significantly more PDGFRα^+^/p-β-catenin^+^ cells than Ctrl mice. Scale bar = 30 μm. Data are presented as minimum to maximum. *n* = 7 per group. ^**^p < 0.01 vs Ctrl; unpaired Student’s t-test. **g**, **h** Confocal images and statistic histograms illustrating the co-localization of p-β-catenin with CC1. As shown, VWR mice have significantly more CC1^+^/p-β-catenin^+^ cells than Ctrl mice. Scale bar = 30 μm. Data are presented as minimum to maximum. *n* = 7 per group. ^***^p < 0.001 vs Ctrl; unpaired Student’s t-test

Previous studies have demonstrated that canonical Wnt signaling functions as a potent inhibitor of OPCs differentiation [28]. Activation of Wnt signaling decreases the phosphorylation of β-catenin by glycogen synthase kinase 3β (GSK-3β) and thus prevents β-catenin degradation. Then, β-catenin competes with HDAC1 and/or HDAC2 to interact with TCF7L2 to inhibit OPCs differentiation [21, 28, 29]. To determine if the VWR-enhanced myelination involved Wnt signaling, we assessed the protein levels of GSK-3β, β-catenin and phosphorylated β-catenin (p-β-catenin) in the motor cortex using western blotting. We found that the levels of GSK-3β and β-catenin were not changed in VWR mice (Fig. 5a-c), whereas the level of p-β-catenin markedly increased (Fig. 5b, d). Furthermore, we used immunohistochemistry and found that p-β-catenin-positive OPCs (PDGFRα^+^) and OLs (CC1^+^) significantly increased in VWR mice (Fig. 5e-h).

**Fig. 6:**
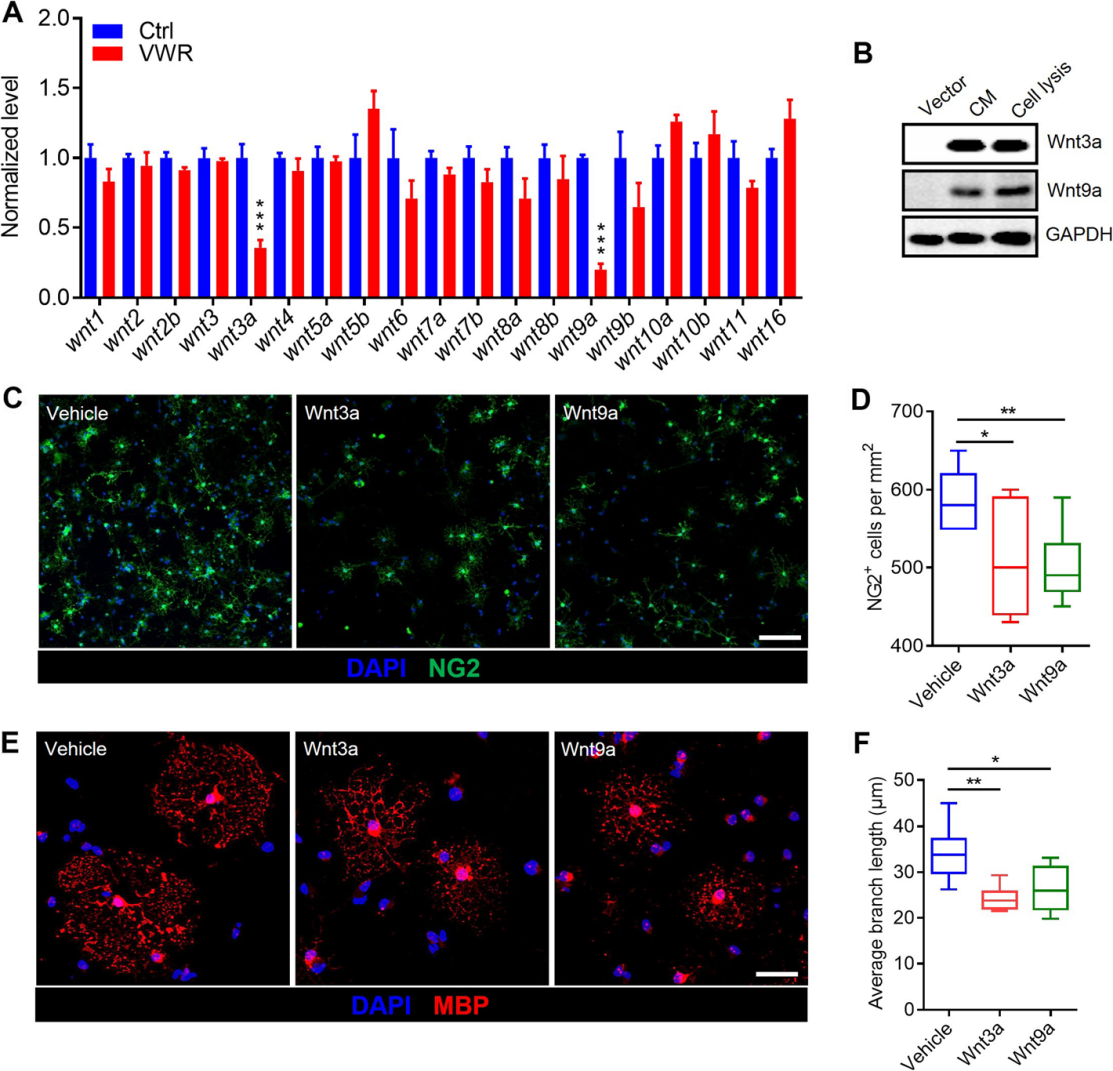
Voluntary wheel running (VWR) promotes myelination via inhibiting the expression of Wnt3a and Wnt9a. **a** The mRNA levels of Wnts in the motor cortex of control (Ctrl) and VWR mice. Data are presented as mean ± SEM. *n* = 6 per group; ^***^p < 0.001 vs Ctrl, unpaired Student’s t-test. **b** Expression of Wnt3a and Wnt9a in HEK293 cells. CM, Cell membrane. **c**, **d** Wnt3a- and Wnt9a-treated OPCs immunostained for NG2 (OPCs). As shown, the number of OPCs decreased upon treatment with Wnt3a or Wnt9a. Scale bar = 20 μm. Data are presented as minimum to maximum. *n* = 7 independent coverslips per condition. ^*^p < 0.05 vs Ctrl; **p < 0.01 vs Ctrl; unpaired Student’s t-test. **e**, **f** Wnt3a- and Wnt9a-treated OPCs immunostained for MBP (OLs). The average branch length of OLs decreased upon treatment with Wnt3a or Wnt9a. Scale bars = 20 μm. Data are presented as minimum to maximum. *n* = 7 independent coverslips per condition. ^**^p < 0.01 vs Ctrl;^*^p < 0.05 vs Ctrl; unpaired Student’s t-test

In order to understand the mechanism through which VWR activates Wnt signaling, we detected the changes of the transcripts of Wnts. We isolated the tissue of the motor cortex using microdissection and conducted qRT-PCR analysis to evaluate the mRNA levels of different Wnts. As shown in Fig. 6, the mRNA levels of *Wnt3a* and *Wnt9a* in the motor cortex was dramatically down-regulated in VWR mice (Fig. 6a). To demonstrate the role of *Wnt3a* and *Wnt9a* in the proliferation and differentiation of OPCs, we expressed and purified *Wnt3a* and *Wnt9a* from HEK293 cell in culture (Fig. 6b). We then developed an imaging assay based on the induction of MBP expression in rat cortex-derived OPCs cultured for 4 days under basal differentiation conditions. We found that Wnt3a and Wnt9a treatment significantly decreased the number of OPCs (Fig. 6c and d) and impaired the efficient differentiation of OPCs into MBP-producing mature oligodendrocytes (Fig. 6e and f).

Taken together, these results suggest that VWR promotes OPCs proliferation and differentiation and ultimately the myelination of the motor cortex, most likely by inhibiting Wnt signaling.

## Discussion

The present study demonstrated that voluntary wheel running promotes the proliferation and differentiation of OPCs and thus enhances the myelination in the motor cortex, and such beneficial effect is probably mediated via Wnt signaling.

It has been documented that central myelination have a huge potential of plasticity [30–32]. In humans, central myelination continues throughout adolescence and adulthood [5, 33, 34]. White matter development correlates with enhanced motor skills, reading ability and cognitive functions [31, 35]. Learning sensorimotor skills, studying a new language, or undertaking working memory training induces structural change in the white matter and promotes oligodendrogenesis and adaptive myelination in the brain [36–39]. On the other hand, some neuropsychiatric diseases, such as amyotrophic lateral sclerosis, bipolar disorder and schizophrenia, are associated with defects in central myelination [40–44]. Social deprivation or chronic stress results in hypo-myelination in the prefrontal cortex, and impairs social interaction ability in mice [45–47]. A previous study in our laboratory revealed that neonatal maternal separation hinders mPFC myelination in rats and impairs mPFC-dependent functions [21]. The present study adds new evidence for the plasticity nature of cerebral myelination, and indicates the role that individual experience has in regulating cerebral myelination.

The existence of an activity-driven myelination has been postulated [31, 32]. Previous studies in rodents show that running skill training or feeding skill training promotes the myelination in related brain regions [48, 49]. Even voluntary running could also promote the differentiation of OPCs in the sensorimotor cortex [50], and accelerate the proliferation of OPCs in the spinal cord [51]. Consistently, the present study demonstrated that OPCs and myelin in the motor cortex were both affected by physical activity like voluntary wheel running.

It is known that physical activity accelerates myelination through an increase in OPCs proliferation and differentiation [48, 52]. The present study found that VWR promoted the proliferation and differentiation of OPCs and the maturation of OLs, and enhanced the myelination in the motor cortex. The VWR-enhanced myelination was associated with improved motor skills. More importantly, we found that the VWR-enhanced myelination was linked with a significant decrease in the level of Wnt3a and Wnt9a, and a significant increase in the level of p-β-catenin, the key molecules in the Wnt signaling pathway. Moreover, stimulation of Wnt signaling pathway with Wnt3a or Wnt9a suppresses OPCs proliferation and differentiation *in culture*. Our findings provide insight into the signaling mechanism underlying the VWR-enhanced myelination in the motor cortex.

In summary, the present study demonstrated that physical activity is highly efficient at promoting myelination by enhancing the proliferation of OPCs and accelerating the generation of myelin in the motor cortex, providing a step forward in understanding the beneficial effects of physical activity on central myelination and its underlying mechanism.

## Supplementary information

**Additional file 1: Table S1.** Source and dilution of primary antibodies.

**Additional file 2: Table S2.** Source and dilution of secondary antibodies.

**Additional file 3: Table S3.** Primers used in the present study.

## Data Availability

The data generated or analyzed are included in this published article.
