# Supplementary material for: Voluntary wheel running promotes myelination in the motor cortex through Wnt signaling in mice
Source: Mol Brain. 2019 Oct 24;12:85. doi: 10.1186/s13041-019-0506-8 (PMC6814131; doi:10.1186/s13041-019-0506-8)
Supplement: Supplementary file 1 — Additional file 1: Table S1. Source and dilution of primary antibodies. [file 13041_2019_506_MOESM1_ESM.docx]

**Table S1. Source and dilution of primary antibodies**

| Target protein or marker | Host species | Source (Product Code) | Dilution |
| --- | --- | --- | --- |
| BrdU | Rat | Abcam (ab6326) | 1:500 |
| GSK-3β | Rabbit | Cell signaling (12456) | 1:1000 |
| β-Catenin | Rabbit | Cell signaling (12475) | 1:1000 |
| Phospho-β-Catenin | Rabbit | Cell signaling (4176) | 1:1000 |
| Olig2 | Rabbit | Millipore (ABN899) | 1:1000 |
| CC1 | Mouse | Abcam (ab2377) | 1:100 |
| PDGFR | Rat | BD Science (558774) | 1:500 |
| NG2 | Rabbit | Millipore (AB5320) | 1:200 |
| MBP | Rabbit | Abcam (ab40390) | 1:1000 |
| GAPDH | Mouse | Thermo Fisher (MA5-15738) | 1:1000 |
| β-Actin | Mouse | Sigma- Aldrich (A5441) | 1:1000 |
