# Supplementary material for: Voluntary wheel running promotes myelination in the motor cortex through Wnt signaling in mice
Source: Mol Brain. 2019 Oct 24;12:85. doi: 10.1186/s13041-019-0506-8 (PMC6814131; doi:10.1186/s13041-019-0506-8)
Supplement: Supplementary file 2 — Additional file 2: Table S2. Source and dilution of secondary antibodies. [file 13041_2019_506_MOESM2_ESM.docx]

**Table S2. Source and dilution of secondary antibodies**

| Target species | Conjugates | Source (Product Code) | Dilution |
| --- | --- | --- | --- |
| Donkey anti-mouse | Alexa Fluor® 488 | Invitrogen (A-21202) | 1:500 |
| Donkey anti-mouse | Alexa Fluor® 568 | Invitrogen (A10037) | 1:500 |
| Donkey anti-rat | Alexa Fluor® 488 | Invitrogen (A-21208) | 1:500 |
| Donkey anti-rabbit | Alexa Fluor® 568 | Invitrogen (A10042) | 1:500 |
| Anti-mouse IgG | HRP | Cell signaling (7072) | 1:3000 |
| Anti-rabbit IgG | HRP | Cell signaling (7071) | 1:3000 |
