# Supplementary material for: Voluntary wheel running promotes myelination in the motor cortex through Wnt signaling in mice
Source: Mol Brain. 2019 Oct 24;12:85. doi: 10.1186/s13041-019-0506-8 (PMC6814131; doi:10.1186/s13041-019-0506-8)
Supplement: Supplementary file 3 — Additional file 3: Table S3. Primers used in the present study. [file 13041_2019_506_MOESM3_ESM.docx]

**Table S3. Primers used in the present study**

| Primers | Sequence (5’→3’) |
| --- | --- |
| *Mbp-F* | ATTCACCGAGGAGAGGCTGGAA |
| *Mbp-R* | TGTGTGCTTGGAGTCTGTCACC |
| *Wnt1-F* | CGAGAGTGCAAATGGCAATTCCG |
| *Wnt1-R* | GATGAACGCTGTTTCTCGGCAG |
| *Wnt2-F* | AGGATGCCAGAGCCCTGATGAA |
| *Wnt2-R* | CGCCTGTTTTCCTGAAGTCAGC |
| *Wnt2b-F* | CCATTACGGTGTTCGCTTTGCC |
| *Wnt2b-R* | CAGCTTCAGGAATCTCCGAACAG |
| *Wnt3-F* | CCGCTCAGCTATGAACAAGCAC |
| *Wnt3-R* | AAGTCGCCAATGGCACGGAAGT |
| *Wnt3a-F* | AACTGCACCACCGTCAGCAACA |
| *Wnt3a-R* | AGCGTGTCACTGCGAAAGCTAC |
| *Wnt4-F* | GAGAACTGGAGAAGTGTGGCTG |
| *Wnt4-R* | CTGTGAGAAGGCTACGCCATAG |
| *Wnt5a-F* | GGAACGAATCCACGCTAAGGGT |
| *Wnt5a-R* | AGCACGTCTTGAGGCTACAGGA |
| *Wna5b-F* | GCTACCGCTTTGCCAAGGAGTT |
| *Wnt5b-R* | CATTTGCAGGCGACATCAGCCA |
| *Wnt6-F* | TTTCCGACGCTGGAACTGCTCC |
| *Wnt6-R* | CCTGACAACCACACTGTAGGAG |
| *Wnt7a-F* | TTCGCCAAGGTCTTCGTGGATG |
| *Wnt7a-R* | TACAGGAGCCTGACACACCATG |
| *Wnt7b-F* | TTCTCGTCGCTTTGTGGATGCC |
| *Wnt7b-R* | CACCGTGACACTTACATTCCAGC |
| *Wnt8a-F* | GGTGACTTGGAAAACTGCGGCT |
| *Wnt8a-R* | CCAAACTGTCCACGAAGAGTCTG |
| *Wnt8b-F* | CGGAGACTTTGACAACTGTGGC |
| *Wnt8b-R* | CTGCTTGGAAATTGCCTCTCCG |
| *Wnt9a-F* | AGTGCCAGTACCAGTTCCGCTT |
| *Wnt9a-R* | GAGATGGCGTAGAGGAAAGCAG |
| *Wnt9b-F* | AGAGAGGAAGCAAGGACCTGAG |
| *Wnt9b-R* | GAGAGCTGCTTCCAACAGGTAC |
| *Wnt10a-F* | GCTCCTGTTCTTCCTACTGCTG |
| *Wnt10a-R* | ATGTCAGGCACACTGTGTTGGC |
| *Wnt10b-F* | ACCACGACATGGACTTCGGAGA |
| *Wnt10b-R* | CCGCTTCAGGTTTTCCGTTACC |
| *Wnt11-F* | GCCTGTGAAGGACTCAGAACTTG |
| *Wnt11-R* | AGCTGTCACTGCCGTTGGAAGT |
| *Wnt16-F* | CCCTCTTTGGCTATGAGCTGAG |
| *Wnt16-R* | GGTGGTTTCACAGGAACATTCGG |
| *GAPDH-F* | CATCACTGCCACCCAGAAGACTG |
| *GAPDH-R* | ATGCCAGTGAGCTTCCCGTTCAG |
